# Touchscreen Tablets: Coordinating Action and Perception for Mathematical Cognition

**DOI:** 10.3389/fpsyg.2017.00144

**Published:** 2017-02-08

**Authors:** Carolien A. C. G. Duijzer, Shakila Shayan, Arthur Bakker, Marieke F. Van der Schaaf, Dor Abrahamson

**Affiliations:** ^1^Faculty of Social and Behavioural Sciences, Education and Learning, Utrecht UniversityUtrecht, Netherlands; ^2^Faculty of Science, Freudenthal Institute, Utrecht UniversityUtrecht, Netherlands; ^3^Graduate School of Education, University of California, Berkeley, BerkeleyCA, USA

**Keywords:** attentional anchors, touchscreen tablet, mathematics, proportional reasoning, sensorimotor interaction

## Abstract

Proportional reasoning is important and yet difficult for many students, who often use additive strategies, where multiplicative strategies are better suited. In our research we explore the potential of an interactive touchscreen tablet application to promote proportional reasoning by creating conditions that steer students toward multiplicative strategies. The design of this application (Mathematical Imagery Trainer) was inspired by arguments from embodied-cognition theory that mathematical understanding is grounded in sensorimotor schemes. This study draws on a corpus of previously treated data of 9–11 year-old students, who participated individually in semi-structured clinical interviews, in which they solved a manipulation task that required moving two vertical bars at a constant ratio of heights (1:2). Qualitative analyses revealed the frequent emergence of visual attention to the screen location halfway along the bar that was twice as high as the short bar. The hypothesis arose that students used so-called “attentional anchors” (AAs)—psychological constructions of new perceptual structures in the environment that people invent spontaneously as their heuristic means of guiding effective manual actions for managing an otherwise overwhelming task, in this case keeping vertical bars at the same proportion while moving them. We assumed that students’ AAs on the mathematically relevant points were crucial in progressing from additive to multiplicative strategies. Here we seek farther to promote this line of research by reanalyzing data from 38 students (aged 9–11). We ask: (1) What quantitative evidence is there for the emergence of AAs?; and (2) How does the transition from additive to multiplicative reasoning take place when solving embodied proportions tasks in interaction with the touchscreen tablet app? We found that: (a) AAs appeared for all students; (b) the AA-types were few across the students; (c) the AAs were mathematically relevant (top of the bars and halfway along the tall bar); (d) interacting with the tablet was crucial for the AAs’ emergence; and (e) the vast majority of students progressed from additive to multiplicative strategies (as corroborated with oral utterances). We conclude that touchscreen applications have the potential to create interaction conditions for coordinating action and perception into mathematical cognition.

## Introduction

Educational theory should offer valuable heuristics for designing applications that foster students’ conceptual learning. However, these theories have by and large focused on learning-with-*paper* rather than learning-with-*technology* (Papert, 2004). This theory-to-practice gap is particularly acute in the case of touchscreen tablets: Whereas tablets offer a breakthrough in human-computer interaction by way of enabling direct multi-touch manipulation of virtual objects, educational research is still scarce on how performing motor actions can contribute to the development of conceptual knowledge (Glenberg, 2006; Marshall et al., 2013; Abrahamson and Bakker, 2016). Even when researchers do engage students in multimodal interaction, where action and perception are elicited as cognitive entry into target concepts, these actions and perceptions are rarely studied via multimodal learning analytics (Worsley and Blikstein, 2014). Consequently, critical data are lost on how action and perception may lead to more advanced reasoning. In the current study we investigated how students could benefit from engaging with an interactive tablet application designed to foster mathematical reasoning through the development of new sensorimotor coordination.

**FIGURE 1:**
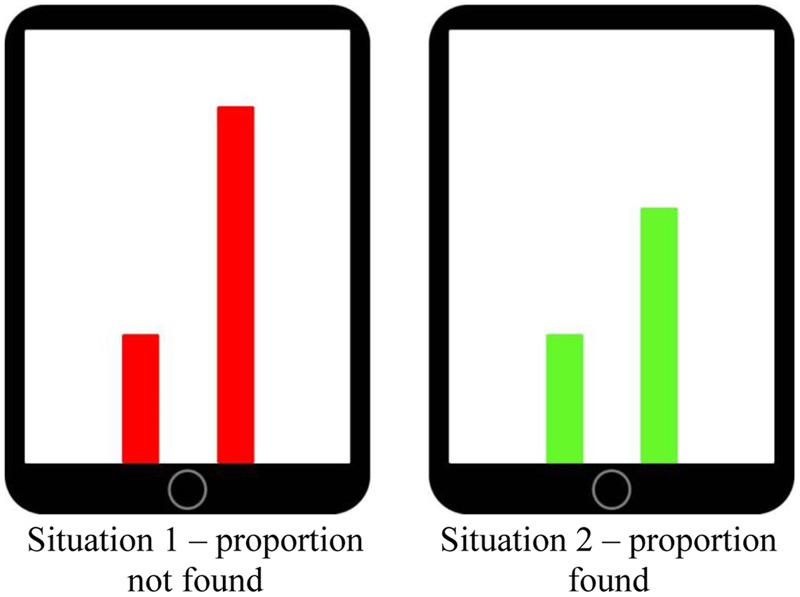
**Representation of the MIT-Ext colored bars.** Both bars have to be moved in parallel motion. Once the right proportion is found (example: pre-set proportion 1:2), the bars will turn green.

Investigating multimodal learning could be especially beneficial in those learning domains in which students are known to experience severe difficulties. Proportional learning is one such area. It could be that students’ difficulty with developing proportional reasoning lies not so much with the mathematical concepts *per se* as much as with their conventional presentation, which is as symbolical expressions of quantitative relations. Symbolic presentation of mathematical concepts, particularly without guiding students in the appropriate multimodal animation of the symbols, is liable to elicit inappropriate understandings, for example it may evoke additive routines where multiplicative solutions are needed. In the current study we use an interactive touchscreen tablet application (MIT-Ext), an extended version of the *Mathematical Imagery Trainer for Proportion* (MIT-P; Reinholz et al., 2010; Abrahamson et al., 2011) that was inspired by arguments from the theory of embodied cognition that mathematical concepts are grounded in sensorimotor schemes (e.g., Varela et al., 1991). In this application students move their fingers up and down along two vertical bars to try and make the bars green. They will be green, rather than red, only when the respective heights of the bars relate by a preset proportion, such as 1:2, that is initially unknown to the students (see **Figure 1**). These physical movement patterns students learn to enact could potentially create opportunities to ground what will become the target mathematical content of proportionality.

**FIGURE 2:**
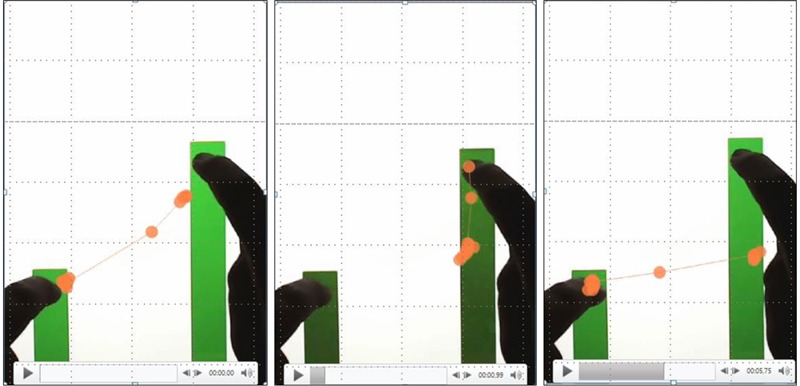
**Three consecutive video stills overlaid with eye gaze data, showing the occurrence of a perceptual triangle, whereby the student looks at the top of the short bar, top of the high bar and halfway up the high bar**.

The current study was designed to investigate the emergence of sensorimotor schemes as students engage in a MIT-Ext task. We hypothesized that while students’ hands move the bars at a constant ratio, their eyes will follow dynamical patterns. These patterns are called attentional anchors (AAs) – psychological constructions of new perceptual structures in the environment that people invent spontaneously as their heuristic means of guiding effective manual actions for managing an otherwise overwhelming task (Liao and Masters, 2001; Hutto and Sánchez-García, 2015; Abrahamson and Sánchez-García, 2016). **Figure 2** demonstrates an AA that occurred frequently in the empirical data.

Prior studies from this research program showed that throughout the task, students often looked at specific parts of both bars and their eyes moved in patterned sequences among these locations. The conjecture arose that these perceptual behaviors consistently predicted students’ conceptual transition from additive to multiplicative strategies (Shayan et al., 2015; Abrahamson et al., 2016). In the current study we examined in detail the process of constructing AAs so as to determine how these perceptual structures facilitated students’ motor actions in accord with the task demand that is, moving the virtual objects while keeping them green. In particular, we describe how the coordination of action and perception stimulated students’ progression from additive to multiplicative solution strategies. We articulated the following two research questions to guide this new line of inquiry:

(1) What quantitative evidence is there for the emergence of AAs?
(2) How does the transition from additive to multiplicative reasoning take place when solving proportion tasks in interaction with the touchscreen tablet app?

In the next section we focus on the theoretical rationale and design methods for investigating embodied-interaction technologies for learning mathematics, and in particular learning proportions.

## Theoretical Background

### Proportional Learning

For primary school students proportion is a notoriously difficult domain of mathematics. In the Dutch school system, the domain “proportions, fractions and percentages” enters the school curriculum in the late elementary grades. Students have to meet particular standards related to ratio and proportion. These standards are set in the so-called Reference Levels Arithmetic (CITO, 2013) and are assessed by a student tracking system and in national examinations (Boswinkel and Schram, 2011). Before the age of 12, Dutch students have to get a sense of the structure and consistency of quantities, whole numbers, decimal numbers, and percentages. Moreover, they should be able to do some (context-bound) calculations with those mathematical objects (CITO, 2013). During the teaching of proportion there is an emphasis on working with a ratio table, which either is given to students or they must recognize when it might be useful (Van Galen et al., 2005). As such, there is a large emphasis on applying learned rules and strategies instead of developing a deep understanding of proportion.

Essentially, proportional learning involves understanding the multiplicative part-whole relations between rational quantities. This means that a change in one quantity is always accompanied by a change in the other, and that these changes are related by a constant multiplier (Piaget and Inhelder, 1966/1977; Lamon, 2007; Boyer and Levine, 2015). Proportional reasoning and the ability to conduct multiplicative operations can be seen as an important precursor for virtually all other mathematical content, including concepts such as ratios, fractions and linear functions (Karplus et al., 1983; Vergnaud, 1983; Lesh et al., 1988; Bakker, 2014). Despite the paramount importance of proportionality, mastering it remains a challenge for school curriculum (Tourniaire and Pulos, 1985; Lamon, 2012). In particular, students experience difficulty in developing fluency with proportions that build upon – yet are differentiated from – simpler non-multiplicative concepts (e.g., additive constructions), notations, terminology, and procedures (Karplus et al., 1983; Tourniaire and Pulos, 1985; Lamon, 2007; Fernández et al., 2012).

Students’ progression from additive strategies to multiplicative strategies can be seen as a central component of their growing proportional understanding. Additive and multiplicative strategies are theorized in different ways. The current study follows the work of Carpenter et al. (1999), Van Dooren et al. (2010), and Abrahamson et al. (2014), in eliciting the sequences discernible in the students’ emerging proportional learning. Additive strategies on the one hand wrongly focus on the additive differences between components of the ratio (1:2 = 3:4 because 1 + 1 = 2 and 3 + 1 = 4) and on the other hand correctly on repeated addition (1:2 = 3:6, because 1 + 1 = 2 and 3 + 3 = 6), while multiplicative strategies draw on the internal ratio of similar units and apply these to other units (1:2 = 3:6, because 2 = 2^∗^1 and 6 = 2^∗^3).

With respect to the development of proportional reasoning a crucial question then is how students *ground* multiplicative conceptualizations of ratio in additive conceptualizations of proportions (Abrahamson et al., 2014) and how this can be supported by making use of interactive touchscreen tablet applications (e.g., embodied learning tasks).

### Embodied Cognition as a Theory for Mathematical Cognition

In its most fundamental form embodied cognition theory states that the mind, body, and its surrounding environment are highly interrelated, and hence, mutually dependent upon each other (Wilson, 2002; Anderson et al., 2012). In this view, human cognition is deeply rooted in the body’s interactions with its physical environment, where (motor) action, perception and cognition are intricately linked, and reasoning consists of reproducing fragments of embodied experiences (e.g., Lakofff and Nùñez, 2000). This opposes views of early mainstream cognitive science epistemology where the mind is seen as an information processing system, operating completely separately from the body’s sensorimotor systems. Per that view, reasoning (including mathematical thought) is non-bodily, timeless and universal, and the formation of concepts is not restricted by physical realities. And yet proponents of the embodiment view conceptualize, cognitive processes and (mathematical) concepts not as abstract but rather as fully embodied, emergent phenomena (Núñez et al., 1999).

Many studies have provided empirical evidence for the embodied nature of mathematical cognition, including the role of the body in appropriating mathematical concepts. For example, in their study on students’ gestures and the embodied knowledge of geometry, Kim et al. (2011) investigated how gesturing facilitated the emergence of mathematical knowledge, by embodying the multisensory properties underlying geometrical concepts. They found that students’ gestures influenced their thinking about geometrical concepts. Moreover, as the geometrical concepts became more complex, the gestures the students deployed became more complex as well, indicating an intricate relation between gesturing and mathematical knowledge formation. Similar results with respect to the embodiment of mathematical thinking and learning were found by Wright (2001), Broaders et al. (2007), and Alibali and Nathan (2012). Another study by Lozada and Carro (2016), investigating Piagetian conservation tasks in students, found that making students active participants in the transformation process, instead of letting them merely observe the same phenomenon, would help them recognize quantity invariance. These studies, among others, suggest that cognition can be a direct consequence of sensorimotor experiences of conceptual exemplars, which indicates that there is a formative relationship between bodily experiences and mathematical concepts (Johnson-Laird, 1983; Malinverni et al., 2012). The guiding principle is that even the most abstract mathematical concepts are in fact grounded in sensorimotor experiences (Núñez et al., 1999; Wilson, 2002; Gallese and Lakoff, 2005) and created by the human imaginative mind via a very specific use of everyday bodily grounded cognitive mechanisms, such as conceptual metaphors, analogical reasoning, or fictive motion (Miller and Johnson-Laird, 1976; Núñez et al., 1999; Lakofff and Nùñez, 2000; Wright, 2001). Following this embodiment perspective, it is thus important that students are offered the appropriate embodied experiences from which to construct these key concepts. However, these are rarely included in current educational practices. For example, when solving problems involving proportions such as, “1:2 = 3:[?],” students cannot *experience* the meaning of proportional *equivalence* as indicated by the “ = ” symbol, since they do not have a structured opportunity to enact, visualize, or conceptualize certain number pairs (Abrahamson and Lindgren, 2014).

One promising approach, capable of facilitating the emergence of sophisticated schemes mobilizing mathematical learning and development, are embodied-embedded instructional technologies – including touchscreen tablets – (Black, 2010; Antle, 2013), which incorporate and enable students’ emerging sensorimotor enactments and visualizations of mathematical concepts (Reinholz et al., 2010; Abrahamson et al., 2011). Certain technologies are based on the premise that directing people to move in specific patterns of action may guide and improve comprehension, problem solving, and learning (e.g., Fischer et al., 2011; Antle, 2013). As such, students can develop pre-symbolical mathematical cognition by engaging in embodied activities that create the right opportunities to build particular action–perception schemes related to proportions. In particular, we present an example of a learning environment designed with the intention that students first develop proportional sensorimotor schemes and later progressively formalize these schemes in the form of mathematical discourse.

As such, by coordinating action and perception students could move from informal goal-directed motions to more formal mathematics, following a concurrent shift from additive toward multiplicative reasoning. Thus the design and evaluation of an interactive technological device for mathematical learning created an empirical context to pursue broader research problems pertaining to the cognitive process of developing quantitative proportional reasoning. Here we are interested in the interplay between action and perception when students work on the touchscreen application described above. Using eye-tracking technology, we evaluate the construct of AAs and its explanatory power to illuminate hidden processes in our findings related to students’: (a) dynamical patterns in visual attention to the objects; (b) hand movement; and (c) reasoning following changes in visual attention.

### Eye-Tracking to Identify Attentional Anchors

An AA, in essence, is an action-oriented perceptual configuration overlaid onto a problem space (e.g., the nearby environment to which people guide their attention). It can take many forms, depending on the properties of a task and the domain in which the task is going to be carried out. For example, a juggler might imagine a geometrical structure (e.g., a rectangle) hovering in the air in order to coordinate his actions. Accordingly, an AA can be seen as a real or imagined object, area, or other *aspect* or *behavior* that co-exists in a person’s perceptual manifold. In other words, AAs can be thought of as a geometrical form overlaid onto the perceived world and functioning as a tool by which one could coordinate their sensorimotor actions (Liao and Masters, 2001; Abrahamson and Sánchez-García, 2016).

Abrahamson et al. (2016) hypothesized that AAs are constructed and used for motor-action coordination when solving the MIT-Ext tasks. They suggest that the AA play critical roles in achieving both the activity’s primary goal of performing the motor action per task specifications and the secondary goal of mathematizing the physical solution strategy. With respect to the tasks used in our study, from an embodiment perspective, there is the assumption that students develop action-perception coordination schemes to tackle the target problem. We expect students to act out goal-directed movements while looking at mathematically relevant areas in the touchscreen task (i.e., top of the bars and halfway the tall bar). Moreover, since the goal of the task is largely unknown for students at the start of the task, it is expected that students first deploy exploratory haphazard eye-movements, and thereafter, when patterns and task-goals are becoming clear, deploy more deliberate and patterned eye-movements directed at the task relevant areas (Haider and Frensch, 1999; Rayner, 2009).

In the present study we want to further investigate the interaction of action-gaze-reasoning behavior by looking into the eye-measures, including fixation count, fixation duration and scan path of the AA patterns, as well as the timing of the AA patterns and how all these relate with the effective solution strategies. In order to elucidate these (mainly) implicit processes, eye-tracking measures are supplemented with concurrent thinking-aloud transcripts (Van Someren et al., 1994). We assume that the combined use of both methodologies will provide us with a more detailed understanding of the hidden and fine-grained aspects of a participants’ perceptual and cognitive processing (Van Gog et al., 2005; Rayner, 2009; Hyönä, 2010; Lai et al., 2013; Van Gog and Jarodzka, 2013).

## Materials and Methods

### Participants

Forty-five fifth- and sixth-graders from five elementary schools in the Netherlands voluntarily participated in the study. The schools were all denominational, where families were predominantly white and from middle class backgrounds. Seven participants were excluded from the analysis, due to technical problems; four had incomprehensible audio, two had unclear dark video, and one had mis-calibrated distorted eye-measures. The 38 remaining participants were included in the analyses (21 male, 17 female; *M*_age_ = 135.37 measured in months, *SD* = 8.37). Before data gathering commenced, the ethical committee board of the faculty of Social Sciences at Utrecht University approved the study (2015). Additionally, informed consent was obtained from the legal guardians of all students involved.

### Materials

#### Task in MIT-Ext

**FIGURE 3:**
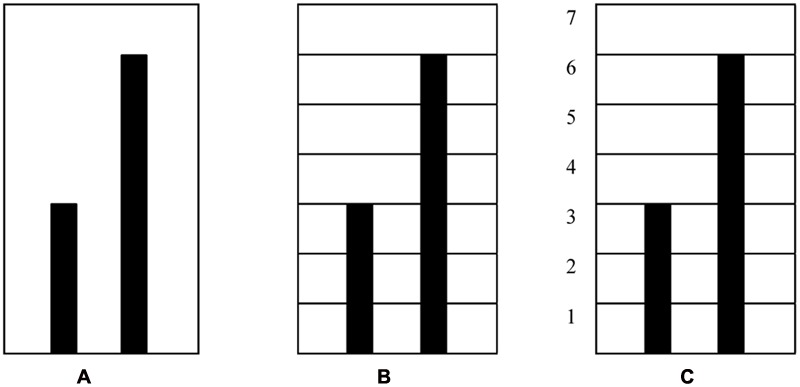
**Schematic representation of the three phases within the task: (A)** pre-set proportion 1:2, blank screen; **(B)** pre-set proportion 1:2, grid; **(C)** pre-set proportion 1:2, grid supplemented with numbers.

The task in MIT-Ext consists of two colored vertical bars. For each bar a student can use their index finger to move the bars up and down. Moving the bars in parallel motion changes the color of the bars along a gradient between red and green. The bars can be set at a predefined ratio (e.g., 1:2, 2:3, 3:4, *etc.*). The bars will *only* turn green when the student finds the *correct* proportion. For example, with the pre-set proportion 1:2, the right bar (RB) has to be twice as high as the left bar (LB). In order to keep the bars green, one has to move their fingers at a pace relative to this pre-set proportion. The aim of the task for the participant is to find *the mystery rule* that causes the bars to turn green (i.e., the pre-set ratios). Eye-tracking technology was added for multimodal data-gathering. MIT-Ext exists of an interface that allows the user to pre-set multiple tasks. The present study included one task, with a pre-set proportion of 1:2. The task consists of three phases, wherein after the first phase, in which the screen is plain white, symbolic artifacts are being added onto the environment (i.e., a grid in phase 2 and a grid supplemented with numbers in phase 3) intended to scaffold a learner’s conceptual understanding of proportions. For a schematic overview of the task and the included phases, see **Figure 3**.

The students were consistenly guided through the environment (Abrahamson et al., 2011, 2014) by following an instruction strategy (i.e., providing cues, e.g., “Try to make the bars green, and maintain the green bars even when you move your hands”).

#### Eye-Tracking Equipment

Eye-tracking data were collected using a Tobii X2-30, mounted on a stand designed for eye-tracking research with mobile devices (e.g., smartphones, tablets). An external camera captured the scene by making video recordings (including audio) during task processing. These recordings were exported to the Tobii software (Tobii, version 3.3.0) to be integrated with the gaze data.

#### Coding Scheme for Video Data and Thinking-Aloud Transcripts

For the analysis of participants’ video data and thinking-aloud transcripts, a coding scheme was developed. The transcripts were coded on the utterance level and consisted of one dimension *knowledge articulation*, divided over two categories (a) *knowledge content*, and (b) *solution strategy*. The first category, *knowledge content* was developed by Chi (1997). Chi’s coding scheme differentiates between unique contributions (C), repetitions of previous contributions (R) and no problem content at all (0) and as such can be seen as a vital part of *knowledge articulation*. Additionally, since the verbally strong participants might have an advantage over the verbally weak participants (Chi, 1997), it seemed reasonable to differentiate between utterances that were contributions and utterances that were repetitions (of previous contributions), including only the contributions into subsequent analyses.

All the *contributions* of the previous category were coded on the second category, solution strategy (Abrahamson et al., 2014). The second category entailed seven ‘strategy’ codes, being: (1) *pre-additive*, (2) *fixed interval*, (3) *changing interval*, (4) *a-*per*-b*, (5) *a*-per-Δ, (6) *multiplicative*, and (7) *speeds*. Short descriptions of the codes with examples are provided in **Table 1**. In essence, within these solutions strategy codes the development of students’ additive conceptions into a more sophisticated multiplicative framework can be traced, following the literature into proportional learning and reasoning (Carpenter et al., 1999; Misailidou, 2007; Reinholz et al., 2010; Van Dooren et al., 2010), but cannot be seen as an ordinal scale in itself. For example, deploying a “speeds” solution strategy is not necessarily *better* or more *advanced* than performing a “multiplicative” solution strategy.

**Table 1:** Characteristics and examples of the two dimensions in the codebook for the video and verbal data.

Dimension	Characteristics	Description/Example	
Knowledge articulation			
(1a) *Knowledge content*			
*Contribution* (C)	Refers to utterances that are indicative of a student’s emerging proportional reasoning.	‘My right hand has to move faster than my left hand to keep it green.’	
*Repetition* (R)	Refers to repetition of previous contributions.	‘When I move faster with my right hand, it remains green.’ [*repetition of the utterance above*]	
*Null-content* (N)	Contains no problem content at all.	‘Can I start already?’	
(1b) *Solution strategy (from additive to multiplicative reasoning)*^a^			

		**Conceptual strategy**	**Motor action**

*Pre-additive* (1)^b^	Comments are focused on the visual appearance of both bars.	‘Right should be higher than left.’	Random movements, green is being found based on chance.
*Fixed interval* (2)^b^	Students try to maintain a constant spatial interval between both hands/bars.	‘There is a difference of two, so I have to go up two at both bars.’	The difference between both bars is being held constant.
*Changing interval* (3)^b^	Students modify the spatial interval between both hands/bars in order to enlarge the distance.	‘The higher I go, the bigger the distance needs to be.’	The difference between both bars is being enlarged.
*a-per-b* (4)^b^	Student deploys sequential hand-movements, each hand moves up or down according to its respective quota.	‘For every unit left, I go up two unit’s right.’	Both bars descend or ascend at respective constant values.
*a-per-*Δ^c^ (5)^b^	Student deploys a strategy that attends to the interval between the left- and right-bar as it changes with respect to the height of the lower bar.	‘1–2 is one line apart, 2–4 is two lines apart, 3–6 is 3 lines apart.’	When the left bar rises, the right bar rises by one unit more than the previous difference between both bars.
*Multiplicative* (6)^b^	Quantitative statements about the numerical location of one of the bars directly as a product of the numerical location of the other bar.	‘The right bar is twice as high as the left bar.’	A value is determined for the left bar, which is continuously doubled to find the value for the right bar.
*Speeds* (7)^b^	Statements are about the relations between both bars in terms of their respective velocity.	‘My right hand has to go faster than my left hand, in order to keep both bars green.’	Both bars ascend and descend at different constant velocities.

*^a^The given examples are based on pre-set proportion 1:2. ^b^Used ordering of the strategies in brackets, the ordering of the used solution strategies was based on the literature into proportional development (e.g., Misailidou, 2007; Van Dooren et al., 2010; Abrahamson et al., 2014). Furthermore, following Abrahamson and Sánchez-García (2016), ‘speeds’ was interpreted as a simultaneous enactment of the *a*-per-*b* strategy while at the same time can be interpreted as a qualitative indication of the multiplicative solution strategy. ^c^Δ = Magnitude of interval between hands.*

Since these sensorimotor enactments were conveyed in essentially the same dynamical hand gestures (i.e., moving the bars simultaneously while keeping the bars green can be interpreted as an enactment of the *a*-per-Δ solution strategy as well as an enactment of the multiplicative solution strategy), the choice was made to primarily rely on the reasoning utterances of the students, while looking at the video data. Moreover, qualitative observations in previous studies into the same tablet application showed that students’ solution strategies preceded or coincided their motor enactments of these strategies (Shayan et al., 2015, 2017). Short descriptions of these motor enactments can be found in **Table 1**. In addition, in the current study, a *pre-additive* code was also included, which is not directly related to a specific sensorimotor enactment, but instead, has a more exploratory nature. During this pre-additive strategy students search for early clues as to why the bars turn green (Reinholz et al., 2010). For a detailed account – and previous use – of the solution strategies in a similar context, see the study of Abrahamson et al. (2014).

### Procedure

#### Pilot Studies

Two pilot studies were conducted in order to test the methodological outset of the main study. During the first pilot study, the MIT-Ext application and the instruction strategy were tested. Four students (age range: 7–10 years) performed several tasks on the MIT-Ext (set-proportions 1:2 and 2:3). For these students the pre-set proportions were difficult. Based on this first pilot study, and following previous research on embodied mathematical learning (e.g., Reinholz et al., 2010; Abrahamson et al., 2011; Petrick and Martin, 2012) and proportional development (Piaget and Inhelder, 1966/1977; Siegler and Pyke, 2013), it was decided to only include students between the age of 10 and 12 years (grade 5–6). A second pilot study was conducted with four students (age range: 11–12 years). Here the pre-set proportions 1:2 and 3:4, and the instruction strategy were tested. Based on this second pilot study it was decided to set the pre-set proportion for the task to 1:2, and to make the instruction strategy more elaborate in order to ensure consistency.

#### Thinking-Aloud Instructions

Following the standards described by Ericsson and Simon (1993), students were encouraged to think-aloud during task performance in order to connect their gaze-data with their proportional reasoning. They were instructed in two ways: (1) written, in the start screen of the task itself and (2) verbally by the researcher. With respect to the environment, a piece of text was incorporated into the MIT-Ext application, twice (“do not forget: say everything you think, out loud to the researcher”). Moreover, whenever necessary throughout the duration of the task, the researcher instructed the students to verbalize everything that came to their minds.

#### Semi-structured Clinical Interviews

The students took part in individual sessions of approximately 1 h during the day in a separate room at schools. At the beginning of each session students got written instructions on the tablet screen, together with images, explaining how to interact with the app: “You have to move the bars up and down and find the green bars. Try to keep the bars green while moving them.” First students were allowed to explore the environment in order to find *as many greens they could*. During this exploratory phase the researcher did not explicitly ask them to express their thoughts. Only when students found the first green, the researcher asked them to find *more greens.* This first phase roughly took 2–5 min (the time students spent per phase and on the whole task varied considerably between students – range in seconds: [419–1475]). After the first exploration phase the students were probed to articulate their thoughts regarding what they were doing and which actions they were undertaking in order to find the *mystery rule*. Regardless of their rule articulation at the end of the first task phase (i.e., blank screen) the students were asked to move the bars all the way up from the bottom to the top while keeping the bars green. After this first phase the previously mentioned instructional probes were repeated throughout the other task phases (i.e., grid and grid supplemented with numbers) as well, while also encouraging the students to express their thoughts about *why the bars turned green* to gain more insight in their used solution strategies. At the end of the third phase the students again were asked to move the bars all the way up. During the task the researcher repeated pre-formulated sentences, such as: *“Can you find more greens?”* and *“Could you tell me what you are doing right now?”* These consecutive interaction periods primed the interview data for subsequent analyses and comparison.

Eye-movements, screen recordings and concurrent verbalizations were captured during the entire task performance. Verbalizations by the students were transcribed verbatim.

### Data Analysis

#### Verbal Transcripts

The coding of utterances was done with a computer program designed for the coding of qualitative data: MEPA (version 4.10) (Erkens, 2005). Two raters familiar with the task and the materials as well as with the coding scheme scored 7.5% of the transcripts (*n* = 3). Inter rater reliability computed on this subsample of transcripts yielded a Cohen’s κ of 0.88 for the knowledge content category, and a Cohen’s κ of 0.73 for the solution strategy category, which both can be considered good. One rater scored the remaining transcripts.

#### Eye-Tracking Data

**FIGURE 4:**
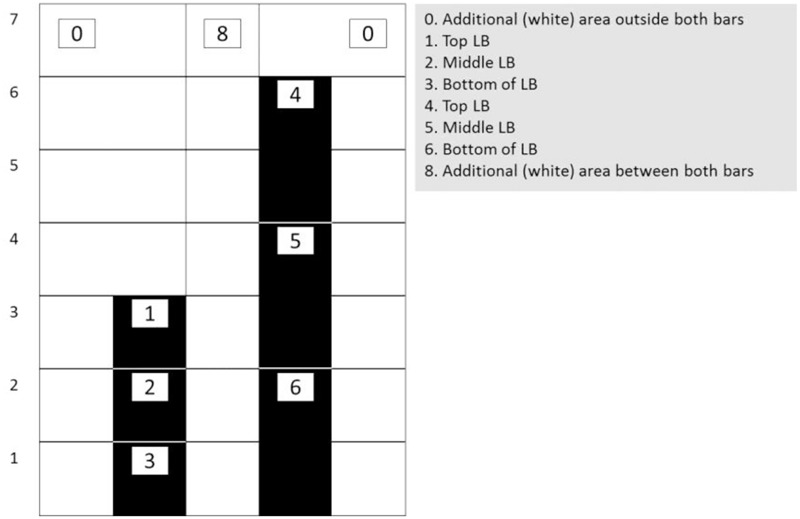
**Schematic representation of the allocation of the eight (dynamic) areas of interest (AoIs) used in current study.** The AoIs on the bars were slightly larger than the bars: 40 pixels on both sides and 40 pixels on top.

For the analysis of participants’ eye-tracking data so-called ‘areas of interest’ (AoIs) were defined. These AoIs were selected based on the mathematical frameworks underlying proportions, which also make sense in the context of the used application (i.e., two bars set a pre-set proportion) (cf., Shayan et al., 2015; Abrahamson et al., 2016). Here it was assumed that students would make sense of the tasks by (1) looking from left to right and vice versa, (2) looking halfway both bars in order to see the shorter bar as halfway the taller bar, and (3) looking at both bars from the top to the bottom and vice versa in order to define the differences between the top of both bars and the bottom of both bars (cf. Fuson and Abrahamson, 2005; Boyer and Levine, 2015). Each bar was divided in three areas of the same size (allowing to gather eye-fixation and gaze data at the top of both bars, halfway both bars, and at the bottom of both bars). These areas would grow and shrink relative to the changes in the bars’ height. Moreover, the area between both bars and the area outside both bars were included as two AoIs as well. **Figure 4** provides a schematic representation of the dynamic AoIs used in the current study.

##### Eye-tracking variables

Based on the literature into problem-solving and expertise development (e.g., Gegenfurtner et al., 2011; Susac et al., 2014), we included four eye-tracking variables in our analysis: (1) the sum of the *fixation counts* within each AA AoI divided by segment time (i.e., time that students seek the mystery rule), (2) the *average fixation duration per visit* by dividing *fixation duration* by *visits in AoIs* (fixation duration was the *total duration by which participants looked at a certain AoI* (in seconds), visits were defined as the successive entering and exiting of an AoI), (3) the *unique visits* by dividing the *visits* by segment time, and (4) the *scan path*, as a count measure, by which participants looked at several AoIs successively.

Besides fixation count, fixation duration, and visits within AoIs it was decided to incorporate the *scan path* because it includes fixation sequences, and gives information about multiple successive fixations and saccades (Holmqvist et al., 2011; Lai et al., 2013). Including the scan path gives insight into the complex patterns of eye movements when processing dynamic stimuli (Jarodzka et al., 2010). In the context of the task this would imply a deeper insight in student’s subsequent eye-movements, and as such could give an indication of the specific eye-patterns that are of interest when solving proportion related tasks. Moreover, when looking at the research of Shayan et al. (2015), being able to quantify the eye-gaze patterns that might have a role in student’s conceptual understanding of proportions is essential to push forward the research in this domain. As such, including the scan path as an eye-measure is insightful in describing the multitude of geometrical variations the AA might hold, and what if any is the role of AAs in the coordination of perception, action and reasoning when performing embodied proportional touchscreen tablet tasks.

##### Pre-processing

In order to process the eye-tracking data the raw gaze data was first filtered with the default Tobii fixation filter (Olsson, 2007). This filter identifies fixation points by a minimum of 5 gaze points grouped within a radius of 35 pixels. Moreover, before going through the recorded data, the Tobii fixation filter applies a correction to missing gaze data points below 100 ms. Using Tobii Studio the gaze data (within segments) was exported to Microsoft Excel. Additionally, using a matlab script the eye-coordinates were converted to the same coordinate system of the hand-coordinates from the apps’ hand movement log files. This was necessary to manually calculate the fixation count and fixation durations of the AOIs with respect to the dynamic height of the bars. Another script was written with python programming language to calculate the fixation duration, visit count, and fixation count (Python, version 2.0; Python, 2015). As such, Python calculated for each time stamp with a fixation point, based on the position of the hands in that timestamp, the associated AoI of the gaze. Moreover, the dynamic track of the gaze data over the AoIs, was also recorded in this program and was returned as the scan path.

#### Analyses RQ 1 What Quantitative Evidence Is There for the Emergence of Attentional Anchors in Terms of Location, Fixation duration, and Scan Paths?

First, for every participant the segments of the eye-data that could be used were defined. It was chosen to focus on the moment between a student started to deploy specific eye-movements indicative of an AA (Shayan et al., 2015) till they articulated their first multiplicative rule (i.e., answering the question: “why do the bars turn green?”). Based on these segments, for every participant the *fixation count, fixation duration*, unique *visits* per AoI, and the *scan paths* over the AoIs were calculated. Descriptive statistics (i.e., frequencies and percentages) are reported to show which AoIs are attended to in terms of count, duration and visits. Next we looked at the scan path and calculated the most frequently occurring gaze sequences. This was done in two ways: (1) time-based, and (2) event-based. For the time-based method, per participant, all occurring patterns were divided by the same participant’s time on task (in seconds). Subsequently, for each occurring pattern these values were added. As a consequence, every pattern got a score indicative of their frequency of occurrence in the sample. As a result, the five patterns with the highest score were picked and included. For the event-based method, per participant, the five most occurring patterns were located. Per participant, the pattern that occurred most got a score of five, the pattern that occurred second most got a score of four, until the least occurring pattern of the five most occurring patterns got a score of one. Subsequently, for each occurring pattern these values were added. As a consequence, every pattern got a score indicative of their frequency of occurrence in the sample. Again, the five patterns with the highest score were included.

#### Analyses RQ 2 How Does the Transition from Additive to Multiplicative Reasoning Take Place When Solving Proportions Tasks?

This second research question is being addressed by analyzing the interaction transcripts in MEPA, to gain a deeper insight into the transitions between the seven different solution strategies in order to detect whether students might show a progression toward more advanced strategies. As such, a lag-sequential analysis was done in MEPA to extract the transitions between solution strategies per participant. Since only the transitions between certain phases were of interest, all repeated consecutive solution strategies were excluded. For example, when a student mentioned the *a*-per-*b* solution strategy twice or more (as a unique contribution) directly after each other, this was changed to mentioning this solution strategy only once. Subsequently, frequency transition tables between all possible combinations of solution strategies were analyzed. Significant transitions are calculated based on a comparison between the observed frequency transition table and an expected frequency transition table where all expected transitions were defined. The values of both tables are then compared to each other to see whether the found transitions in the sample significantly deviates from those transitions one would expect based on chance. Furthermore, since the literature suggests that a transition from additive toward more multiplicative strategies are important indicators for proportional reasoning (e.g., Carpenter et al., 1999; Reinholz et al., 2010; Van Dooren et al., 2010), an aggregated file was made of the initial seven solution strategies into four overarching components, being: (1) *pre-ratio* (or proto-ratio), mostly incorrect strategies (e.g., students keep the distance between both bars fixed; 1:2 = 3:4) (the former pre-additive and fixed distance reasoning), (2) *additive* (the former *a-*per*-b*, and *a*-per-Δ reasoning), (3) *multiplicative* (multiplicative reasoning), and (4) *speeds* (the former change and speeds reasoning, which can be seen as a qualitative account of quantitative multiplicative reasoning). With these four categories it is possible to elucidate students’ transitions from additive to multiplicative frameworks.

## Results

First, video and gaze data were inspected to identify the emergence of AAs. Based on qualitative inspections of video data, the exploratory study of Shayan et al. (2015) already showed that students tend to direct their gaze toward the top of the LB, top of the RB and halfway the RB (length of the LB on the RB), either distinctly or supplemented by separate switches in between those. This focus emerged without explicit instruction. This distinct eye-gaze pattern (AoI 1-4-5, see **Figure 2**) can be seen as indication of an AA. The current study adds to these insights by focusing on the moments before stating the rule of the task. Here students show similar visual patterns, indicating that accomplishing such perceptual-motor (eye-hand) coordination, enabled the students to develop strategies by which they kept both bars green. From this moment on, this distinct gaze pattern will be called a *gaze triangle* whenever necessary. Since the gaze triangle seems closely related to conceptual understanding (i.e., students show similar eye-gaze patterns around the moment they find the solution to the tasks), underscoring the assumption that there are critical phases in knowledge development, the first moments a similar AA appeared were located across the sample. Accordingly, segments were made in Tobii Studio, by which the start of each segment reflected the appearance of the AA for the first time. The end of the segment was marked 5 s after stating the rule of the task (e.g., the RB has to be twice as high as the LB). The moment the students show the first AA till they state the rule will be termed as the *critical phase*, whenever necessary. Subsequently, segments were exported in order to use them for data analysis.

### Results Research Question 1

**Table 2** gives information on students’ eye-measures in terms of counts, fixation duration and number of visits in each of the eight AoIs. From this table it can be noticed that especially AoI 1, AoI 4, and AoI 5 are at the core of the students’ attention, forming a gaze triangle. As such, AoI 1, AoI 4, and AoI 5 were more frequently and longer looked at and visited compared to others. These areas were top of the LB, top of the RB and middle of the RB.

**Table 2:** Means and SDs of number of counts, fixation duration (in seconds), and number of visits in the eight AoIs [percentages given between brackets].

AoIs	Eye-measures					
	**Counts**		**Duration**		**Visits**	
	***M* [%]**	***SD***	***M* [%]**	***SD***	***M* [%]**	***SD***

AoI 0	1541.89 [11.74]	1602.93	49.59 [13.58]	53.46	95.74 [14.28]	95.69
AoI 1^∗^	**2680.95 [20.41]**	2844.17	**71.33 [19.54]**	63.51	**126.32 [18.84]**	109.86
AoI 2	500.26 [5.81]	521.75	16.94 [4.64]	16.98	28.32 [4.22]	25.54
AoI 3	274.24 [2.09]	356.49	7.75 [2.12]	10.83	15.74 [2.35]	16.55
AoI 4^∗^	**4335.92 [33.01]**	4417.90	**114.67 [31.42]**	93.76	**183.68 [27.40]**	149.11
AoI 5^∗^	**1382.76 [10.53]**	1219.66	**43.63 [11.95]**	30.01	**69.66 [10.39]**	52.76
AoI 6	499.53 [3.80]	527.94	15.76 [4.32]	15.92	25.53 [3.81]	24.17
AoI 8	1918.92 [14.61]	1562.14	45.30 [12.41]	31.03	125.32 18.70]	93.09

*^∗^Based on qualitative observation of the video data the AoIs indicative of the AA were 1, 4, and 5.*

**FIGURE 5:**
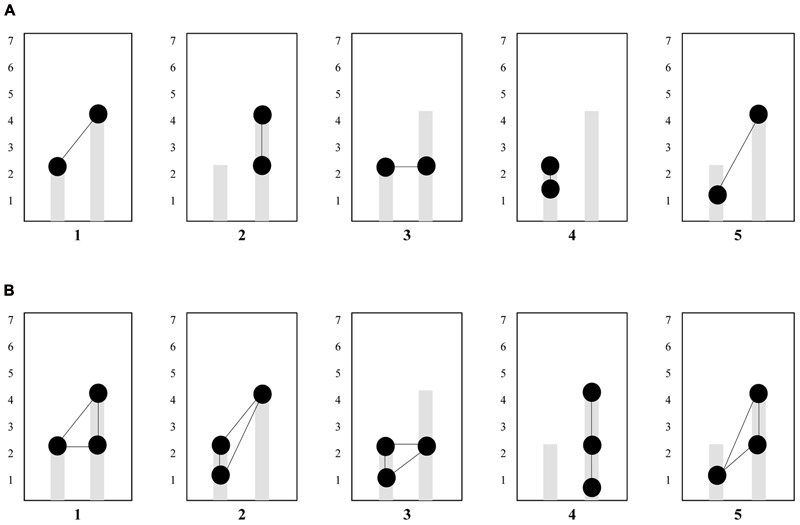
**(A)** Overview of the two-digit gaze-patterns apparent in our sample. Circles connected by lines are representative of the gaze-patterns. Pattern 1 was most prevalent across participants. **(B)** Overview of the three-digit gaze-patterns apparent in our sample. Circles connected by lines are representative of the gaze-patterns. Pattern 1was most prevalent across participants.

Analyses of the occurrence of eye-gaze patterns (i.e., *scan paths*) show that the transition between 1 and 4 (not necessarily in this order) are most common when looking at two subsequent transitions, while the transition between 1-4-5 (not necessarily in this order) are most common when looking at three subsequent transitions. See **Table 3**, for the five most occurring two- and three-digit gaze sequences over the six AoIs. The five most occurring two- and three-digit gaze sequences are visualized in **Figures 5A,B**. Moreover, when looking more closely at the raw data files to see whether these patterns indeed were the most occurring patterns for every student individually, it was revealed that within the two-digit eye-movement patterns, pattern 1–4 was the most occurring pattern for a large portion of the students [60.53%], followed by pattern 4–5 [28.95%], and within the three-digit eye-movement patterns the most occurring patterns were 1-4-5 [73.68%], and 1-2-4 [13.16%], indicating that these eye-gaze patterns indeed were most frequent for most students in the sample.

**Table 3:** The five most occurring (two- and three digit) eye-movement patterns over the six areas of interest (two ways of calculating: using time-based and event-based measures).

	1	2	3	4	5
Two-digit eye-movement patterns					
Time-based (occurrence)	1–4 (67.72)	4–5 (38.81)	1–5 (21.13)	1–2 (10.47)	2–4 (6.34)
Event-based (score)	1–4 (167.50)	4–5 (144.75)	1–5 (82.83)	1–2 (53.33)	2–4 (29.83)
Three-digit eye-movement patterns					
Time-based (occurrence)	1–4–5 (12.87)	1–2–4 (4.32)	1–2–5 (2.98)	4–5–6 (2.50)	2–4–5 (2.21)
Event-based (score)	1–4–5 (167.50)	1–2–4 (95.00)	4–5–6 (57.77)	1–2–5 (52.87)	2–4–5 (46.67)

In sum, manipulating both bars (i.e., performing a situated sensorimotor operatory scheme) in order to keep both bars green corresponded with distinct gaze patterns that students frequently deployed, when progressing through the touchscreen tablet task. Moreover, by acting out goal-directed movements students looked at mathematically relevant areas. In doing so they hooked their initial understanding of proportions to the mathematical structures underlying proportions as this was visually presented in the touchscreen tablet application. Overall, these quantitative results show evidence for the emergence and existence of AAs as was qualitatively observed in the video data.

### Results Research Question 2

**FIGURE 6:**
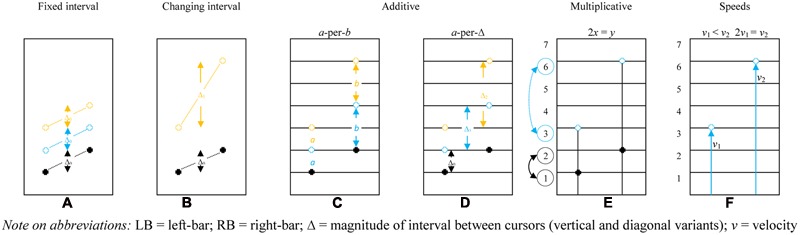
**Adapted from Abrahamson et al. (2014).** Student generated solution strategies for the make-the-bars-green problem (pre-set proportion 1:2): **(A)** fixed interval – maintaining Δ constant regardless of RB-and-LB elevation (incorrect solution); **(B)** changing interval – modifying Δ correlative to RB-and-LB elevation; additive, either **(C)** co-iterate composite units – both LB and RB ascend or descend at respective constant values *a* and *b* (*a-*per*-b*), or **(D)** LB rises by *a* (usually 1), RB by 1 box more than the previous Δ; **(E)** multiplicative – relocating the next green position as a function of the height of only one of the bars (given LB at *x* and RB at *y*, 2*x* = *y*; *x* = ½ *y*), e.g., determining LB *y*-axis value, than doubling to find RB value, then halving for LB, and **(F)** speeds – LB and RB ascend/descend at different constant velocities (*v*_1_ < *v*_2_) or RB velocity is double LB velocity (2*v*_1_ = *v*_2_; *v*_1_ = ½ *v*_2._ LB, left-bar; RB, right-bar; Δ = magnitude of interval between cursors (vertical and diagonal variants); *v* = velocity.

The solution strategies found in current sample are in accordance with the solution strategies outlined in the work of Abrahamson et al. (2014) that was based on video data without eye-tracking technology. **Figure 6** shows a schematic representation of the observed solution strategies in which the strategies and accompanying motor enactments are visualized and explained. Since an in-depth description of those strategies with examples is beyond the scope of this article; the 2014 article gives an elaborate account. In short, the figure shows the six solution strategies. For example, the *fixed interval* solution strategy is shown first. In black you see the first enactment, where the LB is one and the RB is two. Subsequently, the position of the bars change, visualized in blue and yellow (2:3 and 3:4). It is shown that the difference between both bars stays the same (fixed interval), which is incorrect when the pre-set proportion is 1:2.

#### Frequencies and Order of Students’ Solution Strategies

All students in the sample used at least two different solution strategies. Some students used all solution strategies. **Table 4** provides an overview of the strategy occurrence frequencies of the students. Frequencies show that overall the use of solution strategies varies. The pre-additive and multiplicative strategies were used most often. When specifically looking at the solution strategies during the critical phase (i.e., showing the first AA till stating the rule in the first task), it is noticeable that the students used the *a*-per-*b, a*-per-Δ, and speeds strategy the least. **Table 5** shows the overview of transitions between two subsequent codes in the interaction transcript. All statistically significant transitions are presented with *z*-scores between brackets. Closer inspection of the values reveals that the students in our sample more often used a solution strategy further in the sequence after a solution strategy earlier in the sequence than vice versa (106 times vs. 48 times). Especially the transitions from pre-additive solution strategies to fixed solution strategies (10 times, *z* = 3.93), from pre-additive solution strategies to multiplicative solution strategies (20 times, *z* = 3.24), and from multiplicative solution strategies to speeds solution strategies (12 times, *z* = 3.61) seem to occur most.

**Table 4:** Frequencies of solution strategies during the first (pre-set proportion 1:2) task, and during the critical phase.

	Pre-additive	Fixed interval	Changing interval	*a*-per-*b*	*a*-per-Δ	Multiplicative	Speeds	Sum
Tasks								
Task 1	33 (87)	12 (20)	17 (27)	20 (28)	7 (7)	38 (162)	15 (24)	142 (355)
Critical phase	30 (70)	10 (20)	12 (18)	7 (8)	4 (4)	38 (38)	3 (4)	104 (162)

*Absolute frequencies are given (i.e., sum of the students who used that solution strategy). Total amount of used solution strategies are given between brackets. Note that time on task has not been taken into account.*

**Table 5:** Overview of the transitions between two subsequent solution strategies.

Code	1	2	3	4	5	6	7		Total T	Frequency of occurrence
(1) Pre-additive	–	10 (**3.93**)	9 (**2.95**)	1 (-)	2 (-)	20 (**3.24**)	2 (-)		40	44
(2) Fixed	1 (-)	–	5 (**2.75**)	6 (**3.32**)	3 (**3.09**)	4 (-)	0 (-)		18	19
(3) Change	3 (-)	0 (-)	–	5 (**2.25**)	0 (-)	8 (-)	4 (-)		17	21
(4) *a-*per*-b*	1 (-)	1 (-)	1 (-)	–	2 (-)	11 (–)	2 (-)		15	23
(5) *a-*per*-*Δ	0 (-)	0 (-)	1 (-)	1 (-)	–	4 (**2.50**)	0 (-)		4	8
(6) Multi	9 (-)	4 (-)	4 (-)	8 (-)	0 (-)	–	12 (**3.61**)		12	20
(7) Speeds	2 (-)	2 (-)	1 (-)	2 (-)	1 (-)	6 (-)	–		–	61
									106	
Total Transitions	16	7	7	11	1	6	–	48	154	196

*Significant *z*-scores in bold between brackets, *p* < 0.05.*

**Table 6** shows the aggregated solution strategies, and as such the transitions from incorrect toward correct strategies, from additive strategies toward multiplicative strategies, and from multiplicative strategies toward speeds strategies. The first thing to note is that there are significant transitions from (incorrect) pre-ratio solution strategies toward (correct) additive (12 times, *z* = 2.40), multiplicative (23 times, *z* = 3.03), and speeds (16 times, *z* = 3.20) solution strategies. Second, the transition from additive solution strategies to multiplicative solution strategies can be regarded as a significant transition as well (16 times, *z* = 3.43). Finally, as was already discernible in the previous table, there is a significant transition from multiplicative solution strategies to speeds solution strategies (16 times, *z* = 2.34), indicating that the students in the current sample often explicated their quantitative multiplicative insights by a qualitative speeds related account. For example, when a student mentioned a multiplicative solution strategy (e.g., “the RB always has to be half as tall as the LB”), this was more often elucidated by a speeds related solution strategy (e.g., “I have to move my right hand twice as fast as my right hand”) than one would expect based on chance.

**Table 6:** Overview of the transitions between two subsequent aggregated solution strategies.

Code	0	1	2	3		Total T	Frequency of occurrence
(0) Pre-ratio	–	12 (**2.40**)	23 (**3.03**)	16 (**3.20**)		51	51
(1) Additive	2 (-)	–	16 (**3.43**)	4 (-)		20	29
(2) Multiplicative	12 (-)	9 (-)	–	16 (**2.34**)		16	36
(3) Speeds	7 (-)	8 (-)	14 (-)	–		–	61
						**87**	
Total transitions	21	17	14	**–**	**52**	139	177

*Significant *z*-scores in bold between brackets, *p* < 0.05.*

In the next section the findings of the previous sections will be clarified by giving qualitative examples of students’ progression through the task.

### Touchscreen Tablets: A Meeting Place for Action, Perception and Cognition – Two Qualitative Examples

The video data and think-aloud transcripts of two students were chosen to illustrate how touchscreen applications can be a meeting place for action, perception, and cognition. Whereas the previous results sections focus on the presence and frequency of appearance of any AA, and on the order and use of the different solution strategies, we here are integrating all these findings by giving two concrete examples of students progressing through the task. Here we show the variation and commonalities that exist between students. In short, a focus will be on (1) elucidating the findings of the previous section, by giving examples, and (2), elaborating on the cognitive processes (in terms of attention allocation and reasoning) taking place between showing the first AA and stating the rule. As such, these examples will give a fine-grained account of how action and perception are coordinated during emerging proportional reasoning.

**FIGURE 7:**
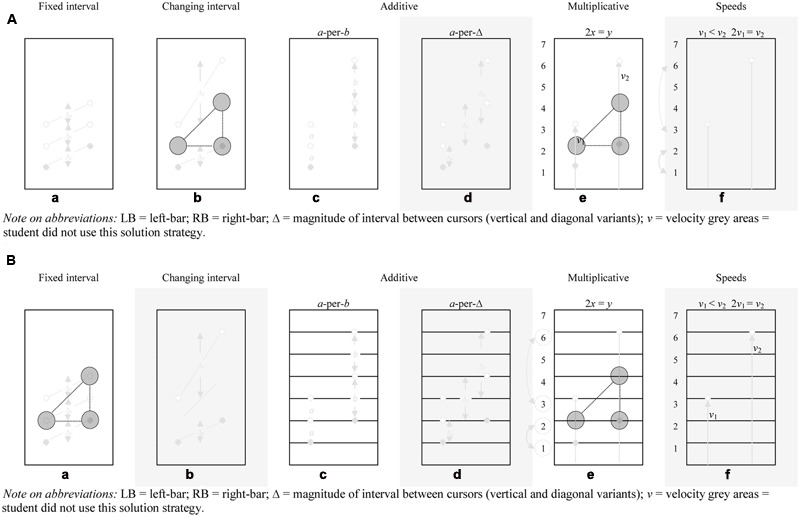
**(A)** (Student 1) Student generated solution strategies for the make-the-bars-green problem (pre-set proportion 1:2): **(b)** First AA occurred during the changing interval solution strategy (time: 00:58) **(e)** Stating the rule – *blank screen* (time: 06:06), **(d,f)** the solution strategies *a*-per-Δ and speeds were not (yet) mentioned by the student. LB, left-bar; RB, right-bar; Δ = magnitude of interval between cursors (vertical and diagonal variants); *v*, velocity gray areas = student did not use this solution strategy. **(B)** (Student 2) Student generated solution strategies for the make-the-bars-green problem (pre-set proportion 1:2): **(b)** First AA occurred during the fixed interval solution strategy (time: 01:52) **(e)** Stating the rule – *grid supplemented with numbers* (time: 13:25), **(b,d,f)** the solution strategies changing interval, *a*-per-Δ and speeds were not (yet) mentioned by the student.LB, left-bar; RB, right-bar; Δ = magnitude of interval between cursors (vertical and diagonal variants); *v*, velocity gray areas = student did not use this solution strategy.

**FIGURE 8:**
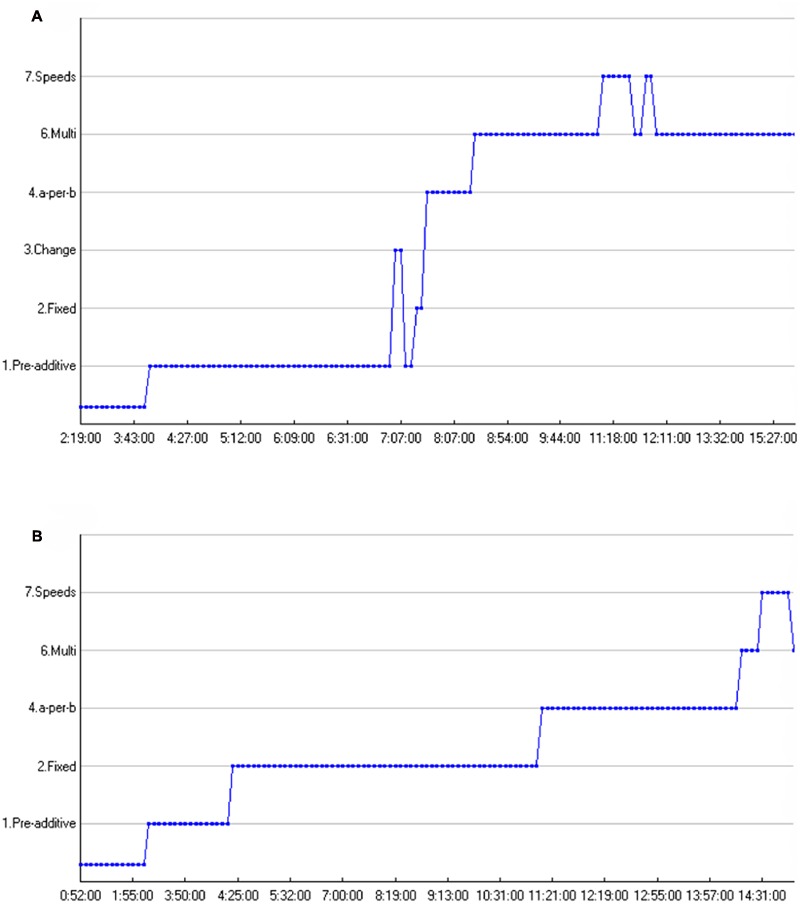
**(A,B)** Sequences of solution strategies, time path on the *x*-axis. **(A)** Student 1 – Solution strategy sequence. **(B)** Student 2 – Solution strategy sequence.

**Figures 7A,B** show the students’ developmental trajectories in terms of appearing AAs during the critical phase. **Figures 8A,B** show the students’ sequences of solution strategies and progression through the entire task. For the first student, see **Figure 7A**, the moment showing the AA for the first time till stating the rule is relatively long. Student 1 articulated a multitude of solution strategies before stating his first multiplicative rule. In this respect, a few moments after the first AA was shown the student stated a strategy related to changing interval. After the articulation of this strategy he articulated a strategy related to fixed interval and a little later gives a qualitative account of the *a*-per-*b* solution strategy. From this latter strategy he gradually progresses into a multiplicative mathematical register (cf., Abrahamson et al., 2014), as shown in the following excerpt:

S1: “It is this piece here [*LB*], which I hold with my left hand [*student moves his left finger up and down the screen*], that should be added over there [*his gaze is focused on the top of the LB while switching to the top of the RB and between the top of the LB and the length of the LB on the RB*].”
[...]
R: “Can you show that to me?”
S1: “Well, for example, it is this part [*difference between both bars*], like this, when that part becomes higher, the bars turn green.”
R: “So, can you explain that?”
S1: “Well, like, that part is just added [*student focuses on the LB*]. For example, this piece [*difference between LB and RB*], actually is doubling the other one [*LB*], so this one [*LB*] is being doubled [*gaze forms a gaze triangle*].”

For his entire solution strategy sequence, see **Figure 8A**. **Figure 7B** shows the developmental trajectory of another student during the critical phase. In general, it took this student longer to state the multiplicative rule than Student 1. This is reflected in the task-phases. Student 1 stated the rule during the first task phase (blank screen), while Student 2 *needed* the symbolic artifacts, not only as a means to enhance, deepen or explain his (naïve) solution procedures, but also to articulate the multiplicative propositions as to why the bars turn green (Abrahamson et al., 2011). In general, Student 2, see **Figure 7B**, had a hard time finding the rule. He articulated many ideas starting with the articulation of some pre-additive rules, the small ‘baby steps’ toward proportional understanding (Reinholz et al., 2010). Subsequently he conveys the *a*-per-*b* solution strategy. From the *a*-per-*b* solution strategy he *slowly* progresses into a multiplicative framework, as shown in the following excerpt, see **Figure 7B**.

R: “What exactly are you doing?”
S2: “I am following the lines.”
R: “Can you explain?”
S2: “[*student’s gaze shifts between the top of both bars*] If this one moves up two, this one moves up one [*still the student shifts his gaze between the top of both bars*].”
[...]
R: “And what are you doing right now?”
S2: “I am sort of, following the lines.”
R: “Can you explain?”
S2: “Yes, whenever…it is starting to double [*intensive gaze shifts between the top of both bars*].”
R: “Ok.”
S2: “Because when this one is at 5 [*looking at the numerals*], I have to move that one [*shifting his gaze between numerals and top of the RB*] to 10 [*here the student uses his thumb to show how the LB is half the RB, adding to his perceptual gaze triangle*].”

In this respect, several minutes have gone by between articulating the *a*-per-*b* solution strategy and articulating the first multiplicative strategy. During these moments he tries to reconcile the grid and the grid supplemented with numbers, interpolated onto the problem space, with his existing strategies, and as such largely shows a similar trajectory as the first student, even though the problem *situation* in which they draw their conclusions is rather dissimilar (i.e., blank screen vs. grid supplemented with numbers). Moreover, with respect to perceptual-sensorimotor coupling, for the second student, while he focuses on the top of both bars extensively, he uses his thumb to assist him in making the gaze triangle, and then states the rule. For his entire solution strategy sequence, see **Figure 8B**.

When looking at the solution strategy sequences of both students, some differences and similarities come to the fore, see **Figures 8A,B**. First thing to stress is that a higher point in the graph does not necessarily mean *better*, as was already discussed in the previous section. However, these graphs do give insight in students’ transitions toward more correct solution strategies, bearing greater mathematical sophistication, and transitions from additive toward multiplicative solution strategies. The first aspect that comes to the fore is that both students mention a speeds solution strategy after a multiplicative solution strategy. Second, both sequences show how the students transition from additive toward multiplicative solution strategies. In this respect, Student 1 and Student 2 show long periods of additive *a-*per*-b* additive reasoning before progressing toward multiplicative reasoning. Certain transitions are illustrative for the entire sample as was showed quantitatively in the previous section. Another important finding (though not visible in the graphs of these two particular students) is that many students “regressed” to lower solution strategies after having first stated a multiplicative strategy. In this respect, *students were prone to explain their initial multiplicative insights in additive terms before progressing toward multiplicative reasoning*.

### Integrative Summary of the Findings

Qualitative and quantitative analysis of the video and eye-gaze data corpus revealed the following patterns:

(1) All students gazed at areas on the screen where there were no particular distinguishing perceptual stimuli *per se*, such as half way along a vertical bar. Believing that these gaze behaviors served the students in better enacting the task’s goal motor-actions, we call these patterns “AAs.”
(2) Whereas individual students invented AAs spontaneously, similar and even identical AAs recurred across the students.
(3) The AAs are related thematically to the mathematical notions instantiated into the activity (top of the bars and halfway along the tall bar can be considered mathematically relevant areas in the touchscreen task).
(4) Within AAs, some Areas of Interest (AoIs) drew greater gaze frequency and durations (see in **Figure 4**).
(5) Comparison across gaze patterns consisting of two AoIs and three AoIs revealed that the most frequent AoI pair was ambiguous with respect to solution insight but the most frequent AoI triad was unambiguous with respect to solution insight.
(6) Students each deployed a variety of solutions strategies.
(7) Transitions between solution strategies were non-random, with strategies that were more correct or bearing greater mathematical sophistication typically occurring after rather than before strategies that were less correct or bearing lesser mathematical sophistication. In particular, solutions tended to progress from additive toward multiplicative rather than vice versa. This would indicate that the touchscreen tablet application for proportions is a means by which students can progress through proportional stages essential for their development of proportional reasoning.
(8) Fine-grained analysis of data from two students revealed the emergence of a multiplicative gaze pattern followed by improved bimanual motor action and then verbal articulation of a successful solution strategy.

All in all, the results demonstrated that participant students’ action, perception, and conceptual understanding developed hand-in-hand through purposeful interaction with a touchscreen tablet application.

## Discussion and Conclusion

The aim of the current study was to understand the micro-process by which embodied interaction with a touchscreen application for proportion may lead to mathematical reasoning. Analysis of the eye-tracking and video data implicated the role of AAs in mediating the coordination of action and perception toward more advanced solution strategies. Two research questions framed the data analyses:

(1) What quantitative evidence is there for the emergence of AAs in terms of fixation count, fixation duration, and scan paths?
(2) How does the transition from additive to multiplicative reasoning take place when solving embodied proportions tasks?

### The Emergence of Attentional Anchors: Inferences from Quantitative Data

The first question was answered by quantifying the eye-gaze patterns that occurred when students interacted with the touchscreen tablet application for proportions (MIT-Ext). These eye-gaze patterns were contemporaneous with first enactments of effective manipulation and prior to verbal articulations of solution strategies. Analyzing these eye-gaze patterns resulted in quantitative evidence of recurrent gaze patterns to screen locations bearing non-salient stimuli or no stimuli at all yet bearing invariant geometric relations to salient dynamical features. As such these eye-gaze patterns apparently guided the students throughout the problem-solving and reflection process. In particular, the AAs are instrumental in passage from task-inappropriate mathematical reasoning (incorrect additive solution) to task-appropriate mathematical reasoning (correct multiplicative solution).

### Transitioning from Additive to Multiplicative Reasoning: The Role of Interaction

It was found that students more often showed a transition from incorrect to correct solution strategies, that is, from additive to multiplicative solution strategies, than vice versa. This indicates that students showed progression toward qualitatively more advanced proportional reasoning at the end of the task than at the beginning of the task. This advancement in reasoning coincided with better coordinated sensorimotor manipulations of the two bars. Case studies illustrated how the emergence of AAs and improved solution strategies co-developed. The dynamics of action-perception-reasoning observed in the current sample speaks for a coherent goal oriented progress in which students use the limited resources available in their working environment to acquire more abstract knowledge in a progressive manner. The path that takes them to reach the goal and to find the ‘rule’ is unique to their experience, yet it shares the necessary building blocks (such as common solution strategies, gaze patterns, etc.) for proportional learning.

These results have several implications, theoretical as well as educational. First, we have presented one example of design-based research in the domain of mathematics education. In addition to the findings outlined here are the possibilities that embodied-design touchscreen applications offer to the education research in general. The current design together with the multi-modal investigating methodology has shed light on the problem at hand (proportional reasoning) from so many different angles that would have remained in the dark otherwise. Without using touchscreen designs it is not possible to study the role of AAs in bimanual coordination. Without the recording of eye-tracking and thinking-aloud to capture students’ perceptual attention and proportional reasoning we would not have found out about the existence of AAs and their correlation with conceptual proportional reasoning. As such, the current study is unique in the sense that it simultaneously studied action, perception, and reasoning and, in doing so, showed how the AA serves as a cognitive pivot in students’ transitions from informal goal-directed motions to more formal reasoning about a mathematical idea. In this respect, the construction of AAs preceded the participants’ articulation of effective manipulation strategies using mathematical terminology. We thus offer first-ever, triangulating empirical evidence in support of claims for the efficacy of the MIT-tablet application. Moreover, based on this study we can be more specific about what aspects of an educational learning environment are crucial for a student’s conceptual development and transfer of knowledge to the task at hand.

Second, given the problems reported about students’ and adults’ proportional reasoning, in particular the persistence of reasoning additively rather than multiplicatively (Lamon, 2007), we find it promising that so many students move from additive to more advanced multiplicative solution strategies in a brief interview session. Within a relatively short period of time the touchscreen tablet application gives students the opportunity to struggle with the core conceptual challenge of proportion, namely that the arithmetic relations among the quantities in a proportional relation are multiplicative instead of additive and that, therefore, the measured differences among corresponding quantities are unequal. As such, the findings of this study support previous research on proportional reasoning. Since it is generally agreed upon that students move from additive to multiplicative reasoning when learning about proportions, we showed that when using an embodied touchscreen application students follow the same developmental course. In general, students in our sample showed additive reasoning and by means of the application changed this to multiplicative reasoning.

### Limitations and Future Directions

The current study did not take differences in verbal ability into account. Since the analyses used largely rely on students’ reasoning utterances, it could be that the verbally weak students are at a disadvantage. In the current study we tried to overcome this problem by (1) taking into account the time students spent on the task, and (2) by not looking into total amounts of utterances students had, but at the transitions between utterances. In this respect, verbally weak students are not necessarily at a disadvantage since we assume that every *new* insight is being articulated (i.e., by means of the instruction strategy that we used consistently within tasks and between students). Nevertheless, future research could incorporate a measure of verbal ability and use it as a covariate to control for any differences between students. Another limitation of the current study is that we only looked at the 1:2 proportion. One could argue that this is a special kind of proportion in the sense that multiplication and division are easily applied to it, and its properties can be more easily visualized than say 2:3. As such, students’ progress through this interactive learning environment may be specific to tasks of finding simple proportions such as 1:2, and to the affordances of this specific touchscreen application. Therefore, transferability of coordination schemes to other proportions needs to be investigated.

Furthermore, it would be valuable to investigate how the occurrence of action-perception schemes relates to other visualizations of the same mathematical domains, or other mathematical domains. In this study we used vertical bars, but we also intend to investigate how students solve tasks when the objects they manipulate are two sides of a two-dimensional shape such as a rectangle. It can be expected that new challenges will emerge, because proportion would then be situated in a multi-directional context, including horizontal as well as vertical movements. As such, work is done on tasks with orthogonal bars looking into the question how strategies students use differ between parallel and orthogonal versions of the task(s). We expect particular pedagogical pay-offs of different representations and mathematical subdomains. Offering students a variation of tasks in which a certain part of that task is being held constant (i.e., the pre-set proportion), can be a powerful source for learning (Runesson, 2006). We also recommend studying pairs of students interacting on the same task. In this way, students are encouraged to communicate about what they are doing (Abrahamson et al., 2011). In this way, re-description (Karmiloff-Smith, 1995) can be promoted and studied. Preliminary results of this line of research are discussed by Abrahamson et al. (2016).

Another important recommendation for future research is to work with the current tasks in more realistic educational settings. Since the touchscreen application is not designed to solely teach about proportions, it is important to design tasks that can support the properties of the application. Also, students worked on the task for a relatively short period of time. How would action and perception evolve when students have more time to work with the touchscreen application? We also recommend to broaden the scope of what is to be learned. The current study focuses on research in the domain of proportional learning. Further research is also recommended on other topics, also beyond mathematics education (e.g., Nemirovsky et al., 1998; Ferrara, 2014). Last, it seems plausible that sub-populations of students benefit more from embodied design than others. It seems worthwhile to study if students with learning disabilities or problems with symbolic language may gain from embodied experiences.

## Conclusion

The discovery of attentional anchors underlying students’ bimanual coordination bears important implications for the design of educational technology. In particular, our refined instrumentation for tracking and visualizing these phenomenological chimera now enables us to reverse-engineer interaction learning. We begin from a mathematical object we wish students to develop, then we construe this object as constituting an AA for some bimanual task, and finally we implicate this task task’s motor-action goals. In a sense, understanding AAs allow educational designers to undo the enactivist evolution from action to perception to cognitive structures, in the service of mathematics learning.

The interview protocol, which was prepared as a research instrument for this study, predicates the experimenter’s clinical interventions at various points along the facilitated activity on the participant manifesting target behavioral criteria. One of these behavior–intervention associations is of particular interest to our research, namely our project to understand micro-processes in guided sensorimotor mathematical inquiry with touchscreen tablet applications. This behavior criterion is students’ effective enactment of a target dynamical motor-action coordination, namely they are moving their hands all the while keeping invariant certain quantitative relations marked by the hands’ momentary locations. As our empirical data demonstrate, although these motor actions manifest multiplicative relations, such as 1:2 ratio, when prompted to articulate their operational strategy the students nevertheless resort to additive structuration. That is, although the dynamical gestalt objectively instantiates an intensive quantity, students explicate their actions in terms of its constituent extensive quantities (specified increments along spatial extensions, such as unitized intervals).

We propose to conceptualize students’ behaviors not as unfortunate regressions but as fortunate opportunities. To begin with, we are assuming that sensorimotor coordinations can emerge as solutions to interaction problems before these coordinations can be articulated logically or modeled mathematically. In a sense, one could argue that the mainstay of our naturalistic and cultural manual skills, such as walking, throwing a stone, or using basic kitchen utensils, come about with little to no conscious, structured reflection let alone discourse. Moreover, we perceive the Mathematical Imagery Trainer activities as creating conditions for students to reflect in retrospect on solutions they have already demonstrated in action. We thus reaffirm our earlier implication of the body as the vanguard of mathematical reasoning (Abrahamson, 2014, 2015): We submit that embodied solutions to coordination problems can exhibit quantitative relations that exceed the individual’s current mathematical knowledge. We further submit that educational technology for sensorimotor mathematical grounding should therefore create conditions for students to enact dynamical instantiations of concepts at the cusp of their conceptual grasp. The teacher’s role is to optimize students’ opportunities for conceptual grounding by challenging and supporting them to explicate their manifest behaviors mathematically. By asking them to coordinate complementary visualizations of their own actions, students may ground multiplicative dynamics in additive conceptions (Abrahamson et al., 2014).

These are early days in our line of investigation. Whereas we hesitate to make causal claims regarding the role of AAs in successful learning with tablet applications, the strong correlations among perception, action, performance, and utterance surely point to promising lines of research, which will hopefully result in creating heuristic design frameworks for educational applications within the field of mathematics. Such frameworks could be informed by ‘embodied design’ (Abrahamson, 2014). In particular, industry informed by embodied design would seek to build touchscreen tablet applications that create opportunities for students to solve motor-action problems designed specifically so as to give rise to targeted proto-conceptual AAs that in turn can assist in reflective abstraction. In our experience the study of what are productive movements and useful coordination schemes in solving tasks that assist in mathematical reasoning is by no means trivial. But with due care, research in this area could inform the work of touchscreen developers building interactive educational applications. In short, touchscreen applications have the potential to be meeting places for action, perception, and cognition.

## Ethics Statement

This study was approved by the ethical committee board of the faculty of Social Sciences at Utrecht University. Informed consent was obtained from the legal guardians of all students involved.

## Author Contributions

All authors contributed extensively to the work presented in this paper. CD and SS took the lead in data gathering and data analysis. All authors were involved in the conception and design of the work; all authors significantly contributed in drafting and revising the work; all authors approve of the version to be published and all authors are accountable for all aspects of the work in ensuring that questions related to the accuracy or integrity of any part of the work are appropriately investigated and resolved.

## Conflict of Interest Statement

The authors declare that the research was conducted in the absence of any commercial or financial relationships that could be construed as a potential conflict of interest.
